# Nanoscale Wetting of Single Viruses

**DOI:** 10.3390/molecules26175184

**Published:** 2021-08-26

**Authors:** Annalisa Calò, Aitziber Eleta-Lopez, Thierry Ondarçuhu, Albert Verdaguer, Alexander M. Bittner

**Affiliations:** 1 Department of Electronic and Biomedical Engineering, University of Barcelona, Calle Marti i Fraquès 1-11, 08028 Barcelona, Spain; 2 Institute for Bioengineering of Catalonia (IBEC), Calle Baldiri Reixac 10-12, 08028 Barcelona, Spain; 3 CIC nanoGUNE (BRTA), Tolosa Hiribidea 76, 20018 Donostia, Spain; a.eleta@nanogune.eu; 4 Institut de Mécanique des Fluides de Toulouse (IMFT), Université de Toulouse, CNRS-INPT-UPS, 2 allée du Professeur Camille Soula, 31400 Toulouse, France; thierry.ondarcuhu@imft.fr; 5 Catalan Institute of Nanoscience and Nanotechnology (ICN2), CSIC and the Barcelona Institute of Science and Technology, Campus UAB, 08193 Bellaterra, Spain; averdaguer@icmab.es; 6 Ikerbasque, Basque Foundation for Science, Pl. Euskadi 5, 48009 Bilbao, Spain

**Keywords:** Tobacco Mosaic Virus (TMV), nanoscale wetting, multifrequency AFM, force reconstruction, amplitude-modulation AM-AFM

## Abstract

The epidemic spread of many viral infections is mediated by the environmental conditions and influenced by the ambient humidity. Single virus particles have been mainly visualized by atomic force microscopy (AFM) in liquid conditions, where the effect of the relative humidity on virus topography and surface cannot be systematically assessed. In this work, we employed multi-frequency AFM, simultaneously with standard topography imaging, to study the nanoscale wetting of individual Tobacco Mosaic virions (TMV) from ambient relative humidity to water condensation (RH > 100%). We recorded amplitude and phase vs. distance curves (APD curves) on top of single virions at various RH and converted them into force vs. distance curves. The high sensitivity of multifrequency AFM to visualize condensed water and sub-micrometer droplets, filling gaps between individual TMV particles at RH > 100%, is demonstrated. Dynamic force spectroscopy allows detecting a thin water layer of thickness ~1 nm, adsorbed on the outer surface of single TMV particles at RH < 60%.

## 1. Introduction

Environmental moisture is a key factor that influences the biological activity of different families of viruses, and, consequently, the spread and the transmission of many virus-mediated infectious diseases. Much work has been focused on this correlation for the case of human viruses. For example, biological assays correlated the seasonality and infectivity of human Influenza A and Norovirus with the total amount of water vapor present in the air, i.e., with the absolute humidity [1,2,3,4]. The infectivity of Rhinovirus-14 has been tested in in vitro experiments and found to be dependent on relative humidity (RH) [5]. The mechanisms through which environmental water vapor affects the spreading of human viruses have not been determined unequivocally; they can be expected to depend on the virus structure at the molecular level [6]. Yang and coworkers proposed that variations in RH modify the pH of aerosols made up of micrometer-sized droplets containing hydrated virus particles and trigger conformational changes in glycoproteins at the surface of enveloped viruses that ultimately affect infectivity [7].

Studies assessing the effect of the environmental humidity on single viruses would attempt to access their shape and characteristic features at the nanoscale in different conditions of hydration. In atomic force microscopy (AFM), such systematic studies have been limited so far by the fact that most investigations on viruses are performed in liquid conditions, which are considered physiological and preserve native structure and biological functionality [8,9,10]. In some cases, collapse of individual viruses has been observed during desiccation, as in the case of bacteriophage φ29 [11].

In this work, we study the wetting of individual adsorbed Tobacco Mosaic Virions (TMV) at varying relative humidities by AFM. The exceptional stability of TMV particles makes them optimal candidates for experiments that need to be performed in air. The characteristic tubular shape with a typical length of ~300 nm and a height of ~14 nm is preserved (the diameter of 18 nm found in bulk virus samples is reduced by radial deformation upon adsorption on surfaces) [12,13]. In TMV, the high mechanical and chemical stability [14,15] is related to the need for this plant virus to “survive” in air and in soil, under highly variable and often adverse environmental conditions [16]. Nevertheless, standard protocols require plants infected with TMV to be inoculated and stored at high RH (80% < RH < 100%) [17,18]. Indeed, transmission in wet conditions, via clouds [19,20], is known for Tomato Mosaic Virus (ToMV), a virus that is practically identical to TMV. These observations suggest single particle studies at varying levels of moisture.

In our experiments on TMV samples performed at high RH, we found a strong contrast between regions containing extended water droplets and the remaining part of the surface whenever we recorded the second excited mode during multifrequency experiments [21,22]. In this work, we present standard AFM topographic images correlated with phase shift images obtained with the second excited mode (Φ_2_), which were collected simultaneously with the topography. Even with optimized multifrequency imaging conditions [12], the quantification of the amount of water adsorbed on the surface of hydrated TMV particles remains challenging and can be barely deduced from a statistical analysis of many topographic profiles collected at various humidity levels. Here we show that dynamic force spectroscopy [23,24] allows detecting a thin water layer, ~1 nm thick, on the surface of single TMV particles at ambient and low RH < 60%. So far, the capability of this technique to detect water layers with sub-nm resolution has been demonstrated for the case of inorganic crystals [25], inorganic surfaces [26], and flat polymer coatings [27] in force reconstruction experiments performed in ambient conditions.

## 2. Results and Discussion

**Figure 1:**
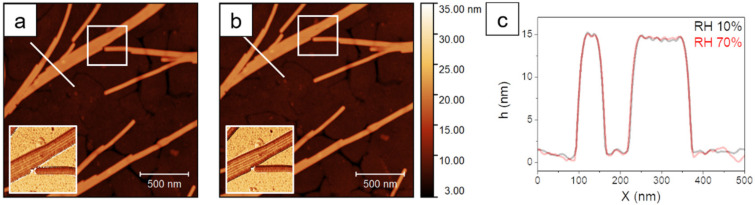
AFM images of Tobacco Mosaic virions on gold at 10% RH (**a**) and at 70% RH (**b**) obtained from standard tapping mode imaging with single frequency excitation. The insets of (**a**,**b**) show Φ_1_ images of the areas enclosed in the white frames. Topographic profiles at the two different humidity levels are shown in (**c**). They refer to the white lines in (**a**,**b**).

Depending on the concentration of virus suspension, on surface pretreatment, and on the adsorption procedure, the coverage of TMV on surfaces can be adjusted in a wide range (see Figure S1 of the Supplementary Materials). Figure 1 shows TMV, adsorbed in rather high coverage on plasma hydrophilized gold, with substantial end-to-end assembly, resulting in rods of microscale length. The helical arrangement of the coat proteins allows for the assembly of gapless and quasi endless rods. Single virions in laterally aligned rods can be distinguished by topographic profiles and phase images. Moving from very low humidity (10%) to high values (70%) does not show changes. While such high coverages are useful for studying, e.g., the protein assembly, we will now focus on very low coverages in order to observe the behavior of single virus rods.

**Figure 2:**
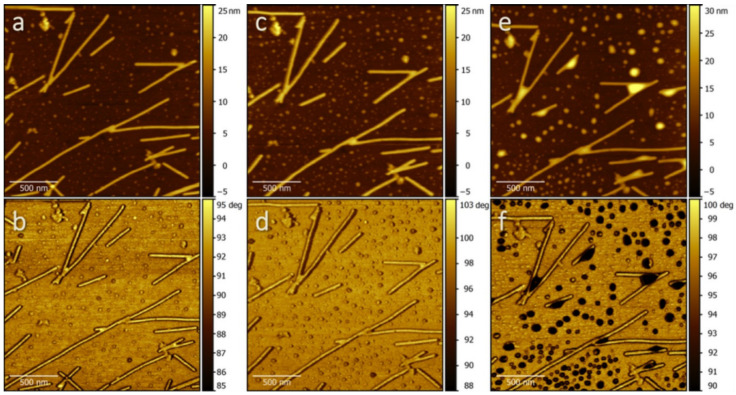
Sequence of AFM topographic images (**a**,**c**,**e**) and corresponding multifrequency Φ_2_ images (**b**,**d**,**f**) collected at RH = 56% (**a**,**b**), 76% (**c**,**d**), and >100% (**e**,**f**).

Throughout our study, we found that the AFM sensitivity towards water imaging improves when we excite the cantilever at two resonant frequencies corresponding to the two first flexural modes, a technique called multifrequency AFM [28]. Figure 2 shows a sequence of standard topographic AFM images (Figure 2a,c,e) and corresponding Φ_2_ images (phase shift of the high frequency mode) (Figure 2b,d,f). Topography and Φ_2_ maps were obtained simultaneously on the same region of the sample containing single TMV particles deposited on plasma-hydrophilized gold. Images were collected at increasing humidity conditions, i.e., at RH = 56% (Figure 2a,b), 76% (Figure 2c,d), and >100% (Figure 2e,f) (see Materials and Methods). Multifrequency images were recorded in the net attractive tip-sample interaction regime for the first fundamental mode [29,30], as inferred from the phase shift of the low frequency excitation (Φ_1_), which always exceeded 90° [12]. The sensitivity of the second mode leads to a much higher contrast than standard measurements based on the fundamental mode (see Figure S2 of the Supplementary Materials) and allows for clear identification of water morphologies for sizes above ~0 nm. Details of the multifrequency experiments are reported in the Section S2 of the Supplementary Materials.

Samples at the lowest humidity level show individual virions and long, linearly aggregated filaments of length above microns and height ~14 nm, on a rather rough surface. On gold surfaces, some virions meet in one point, giving rise to V-shaped structures which are useful to study the water contact angle on the virus. Starting from RH around 76%, the surface roughness appears to increase due to the presence of few additional and/or bigger droplets, which confer contrast to Φ_2_ images (Figure 2c,d), and whose size increases with increasing RH (Figure 2e,f).

First, we investigated the shape of the liquid as revealed by the topographical and Φ_2_ images. The TMV particles act as nucleation sites for water capillary condensation. This allows for interpretation of the TMV/gold surface as a nanostructured surface undergoing wetting [31]. A similar analysis has already been proposed by Alonso et al. using electron microscopy images (STEM and SEM), which do not provide 3D (height) information [32,33].

Assuming equilibrium between liquid water and vapor, and applying the Kelvin equation [34,35], one can estimate the size of water bridges at TMV, in terms of their average curvature radius *r*, in function of the relative humidity RH, as:

(1) $$r=\frac{{\gamma V}_{m}}{\mathrm{RTlnRH}}$$

once *γ* (surface tension), *V_m_* (molar volume), *R* (gas constant), and *T* (absolute temperature) are known. By varying the temperature, we can explore a large range of Kelvin radius values (see Materials and Methods). While at very low humidity only molecular sized cavities should be wetted, water structures with a typical (negative) curvature radius of *r* ≈ −1.2 nm are expected according to the Kelvin equation at RH ≥ 60%. This means that structural features of the TMV surface, i.e., gaps between two coat proteins in the capsid (distance: 2.3 nm), would be completely filled by water at this humidity level. Since this is below the resolution of AFM imaging, no measurable change in the Φ_2_ images is observed for humidity values below 56%. At RH = 76%, the curvature radius of water layers (*r* ≈ −1.9 nm) starts to become comparable with the lateral resolution capabilities of AFM. Interestingly, under these conditions, we start to observe contrast in Φ_2_ images, and the contours of the bigger droplets appear darker (see Figure 2c,d). The wedge formed at the contact between the virion and the surface could be filled completely only at very high RH (>94%, *r* ≈ −9 nm). This leads to water structures with thickness in the range of the TMV size.

Under conditions of oversaturation and water condensation (RH > 100%), we observe water accumulation in positively curved structures along single virions by AFM, which eventually extend to neighboring particles in the V shaped arrangement. This is similar to what one could expect for infectious droplet nuclei in the case of human viruses [4]. At this relative humidity, we also found dark, submicrometric droplets on the gold surface (Figure 2e,f). Kinetically, such droplets should evaporate in less than 1 ms, even close to 100% RH. In our observation time (several minutes), we did not observe changes in the droplets size. This is coherent with the slow kinetics reported in experiments [36]. In our case, the rather rough substrate surface that we found already in dry conditions (RH < 10%) can be responsible for such slow evaporation; it should furthermore function as nucleation promotor, thus stabilizing liquid droplets (see Supplementary Materials Section S3). Other multifrequency images of TMV at different RH are shown in Figure S4 of the Supplementary Materials.

In our experimental conditions, the wetting process was completely reversible, i.e., drying the samples at 25 °C restored the original sample morphology. We exclude any irreversible collapse upon drying [11], a fact that also correlates well with the high mechanical stability of TMV [15,37].

**Figure 3:**
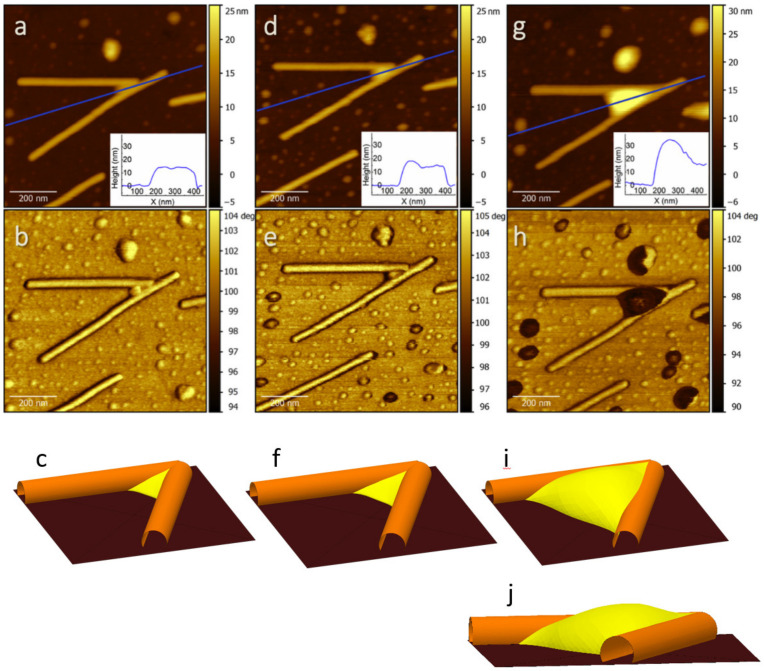
AFM topographic details showing TMV particles in a V-shaped arrangement (**a**,**d**,**g**), and corresponding Φ_2_ images (**b**,**e**,**h**). Surface Evolver simulations were obtained by increasing pressure from negative (**c**), to zero (**f**), to positive (**i**,**j**) values. AFM data correspond to RH = 56% (**a**,**b**), 76% (**d**,**e**), and >100% (**g**,**h**). (**a**,**d**,**g**, inset) Topographic profiles correspond to the blue lines in (**a**,**d**,**g**). A side view of (**i**) is shown in (**j**).

Figure 3 shows a detail of Figure 2 with two linearly aggregated TMV filaments in a V-shaped arrangement. Droplet confinement in such structures (Figure 3g,h) is reminiscent of the corresponding macroscale scenario that we simulated with Surface Evolver under pressure constraint (Figure 3c,f,i,j) [38]. We set the contact angles to 30°, according to the measured values for water on viruses and on gold (see Section S4 of the Supplementary Materials). Details of the simulations are in the Section S5 of the Supplementary Materials).

Interesting details from the simulation under high liquid pressure (corresponding to RH > 100%) include positively curved water structures that extend slightly above the height of the confining virions (see Figure 3i,j). The qualitative agreement between experiments and simulations suggests that the lateral and vertical filling of the area enclosed by two TMV virions by water at increasing humidity (pressure in the simulation), exhibits no anomalous behavior due to nanoscale confinement. This surprising similarity of macroscale and nanoscale contact angles has already been documented for the case of liquid octane nanostructures on organic layers [39]. Topographic profiles along the region where the two virions intersect are reported in the inset of Figure 3a,d,g. For RH > 100%, it gives ~35 nm as maximum height of the confined water.

The strong contrast that we observed in Φ_2_ images between the regions of water accumulation (darker) and the remaining parts of the surface (brighter), which is in the order of expected Φ_2_ differences from numerical simulations [22], allowed us to study the formation of water structures between virions and substrate, with tens of nm typical sizes. Yet, the full characterization of the wetting properties of the TMV tubes, which is essential to understanding their stability in a moist atmosphere, requires going one step further and detecting the presence of water layers wetting the surface of individual TMV tubes. Indeed, thin (one to few monolayers) water films are expected on the highly curved surface of TMV, based on a simple estimation of liquid layers stabilized by long range forces on cylinders [31]. We were unable to detect such wetting layers by AFM imaging. Even in high resolution Φ_2_ images, the surface morphology on top of the virions appeared homogeneous and quite insensitive to RH%, except for the virion contours (see Figure S3 of the Supporting Information).

To detect water adsorption on single virions, we used advanced force spectroscopy tools. Amplitude and phase shift curves (APD curves) were collected on top of individual TMV particles during dynamic force spectroscopy experiments performed at the lowest resonance frequency. Force curves were reconstructed from APD curves according to the Sader–Jarvis–Katan formalism [40,41], and energy dissipation curves were obtained from the Cleveland equation vs. tip-sample distance (*d*) [26,42]. The ability of dynamic force spectroscopy to detect thin water layers on surfaces in ambient conditions is due to the improved resolution in the attractive interaction regime [23]. Details of the experimental method are reported in the Supporting Information Section S6.

**Figure 4:**
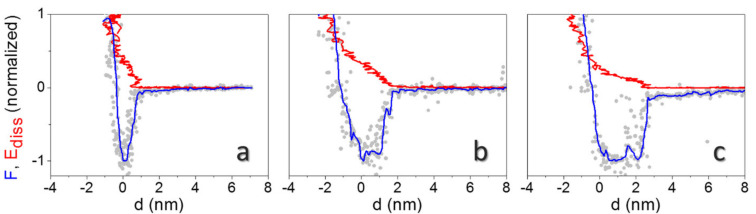
Typical normalized reconstructed F (grey dots: raw data; blue line: data after smoothing) and E_diss_ curves (red line) on top of individual virions at RH < 10% (**a**), 56% (**b**), and >100% (**c**). Curves were obtained from APD curves collected at free amplitude A_0_ = 27 nm. F_ad_ = 2.1 nN (**a**), 0.75 nN (**b**), and 1.1 nN (**c**). E_max_ = 64 eV (**a**), 51 eV (**b**) and 71 eV (**c**).

Typical force and energy dissipation curves (F, E_diss_ vs. *d* curves) on single virions are reported in Figure 4a,b,c at three different environmental conditions. AFM images were collected before the force spectroscopy experiments to localize individual virions, then the cantilever tip was positioned on top of a single virion. AFM images were also collected between each set of APD curves to guarantee no drift had occurred and that the position of the tip compared to the virion did not change during curves collection. Curves are normalized with respect to the minimum in force (adhesion force, F_ad_) and to the maximum dissipated energy in one oscillation cycle (E_max_).

The vertical distance covered in reconstructed force curves, from the beginning of the attractive region up to the onset of mechanical contact (*d* = 0), increases from d < 1 nm at RH < 10% (Figure 4a), to ~2 nm at RH = 56% (Figure 4b), and to ~3 nm at RH > 100% (Figure 4c). These values should approximately correspond to the sum of the hydrated layers on the tip and the surface. Statistical analysis from experiments performed on different virions at the minimum free amplitude (A_0_), to achieve a smooth transition between the attractive and the repulsive regime [29], gives 1.3 ± 0.3 nm, 1.8 ± 0.6 nm, and 3.0 ± 0.4 nm for this distance, respectively. Furthermore, square well-like profiles, where the force shows an abrupt decay followed by an almost constant plateau, stabilize at high humidity levels (see Figure 4c), while approximatively linear force decays are observed in the long range at RH < 10% (Figure 4a).

**Figure 5:**
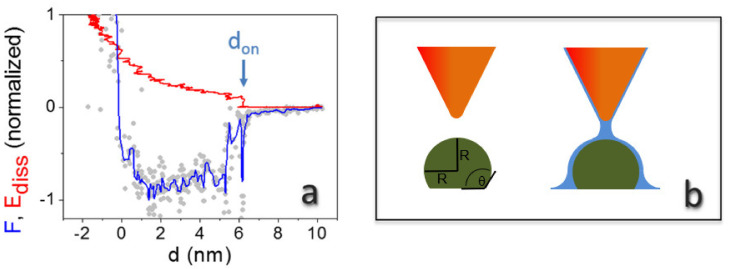
Typical reconstructed F curve (grey dots: raw data; blue line: data after smoothing) and E_diss_ curve collected on top of an individual virion at RH > 100% (A_0_ = 36 nm, F_ad_ = 2.4 nN, E_max_ = 257 eV) (**a**). Sketch of the tip of an atomic force microscope in the proximity of the surface of a virus in dry (left) and wet (right) conditions (**b**). The axial view shows also the water wedge at the interface virion/substrate; the curvature of the wedge is the inverse radius of TMV (*r*^−1^ = R^−1^= (9 nm)^−1^).

Linear decays in force are theoretically predicted when the tip penetrates confined water during approach, assuming a regime of constant vapor pressure [34]. They have been observed on inorganic crystals at intermediate humidity levels [23]. Square well profiles, such as the one shown in Figure 4c, have been observed in many more experiments of dynamic AFM under ambient conditions [23,26]. Here, theoretical expressions for capillary interactions generally fail to describe experimental curves, and a force with a predominantly attractive component independent on distance has been proposed, extending several nanometers above the surface [43]. They have also been observed and modelled for AFM tip nanodispensing [44]. In our experimental conditions, footprints of capillary forces are especially evident at RH > 100%. They are related to the contextual observation of the E_diss_ evolution with *d* (Figure 4c). The E_diss_ increase of ~5 eV, in correspondence with the steep decay in the force at *d* ~3 nm, could indicate a *d*_on_/*d*_off_ mechanism, expected when a capillary neck abruptly forms upon approach at a distance *d* = *d*_on_ and breaks upon retraction at *d*_off_ > *d*_on_ [30]. Santos et al. elucidated that the sudden increase in E_diss_ at *d* = *d*_on_ results from the hysteresis between the approach and the retract path of force curves during single cantilever oscillations [24]. This behavior is even more apparent when blunt tips are used in experiments (see Figure 5a). Here, we clearly observed a jump of ~30 eV in E_diss_ at *d* ≈ 6 nm. The average distance from the onset of the force decay to mechanical contact in curves collected on different virions is 5.6 ± 1.5 nm in this case [44]. The energy involved in the process of formation of a water neck (5–30 eV, depending on the probe’s size) is in the expected range for nanometric tips [24].

The E_diss_ vs. *d* evolution at low and intermediate humidity levels (Figure 4a,b) is different and E_diss_ increases monotonically up to F_ad_. Examples of other curves obtained with blunt tips at different RH levels are shown in Figure S10 of the Supporting Information.

The extracted thickness of confined water at RH = 10% and 56% from dynamic force spectroscopy fits well to a simple estimation based on the Kelvin equation, because the respective distances are related to the neck structure between tip and virion (see Figure 5b). The corresponding value at saturation should correspond to the effective tip curvature radius. Interestingly, the difference between the thickness at saturation and at intermediate RH values (RH = 56%) is in good agreement with topographic data. Topographic profiles on the TMV particles give, in fact, 14.1 nm ± 0.7 nm (Figure 3a) and 15.5 nm ± 0.6 nm (Figure 3g). This confirms that the carefully controlled mild imaging conditions in non-contact AFM can probe surface topography, including water layers, with minimal artifacts [45].

To estimate the thickness of the adsorbed water on virions from reconstructed force curves, one must take into account that water is also present on the tip, which exhibits a SiO_2_-like chemistry. Isobaric X-ray spectroscopy measurements on planar SiO_2_ surfaces gave a water layer thickness of 0.2 to 0.4 nm for RH < 10%, and a rather constant value of ≈1 nm in the 30 to 80% RH range [46]. On the highly curved tip, we expect even thinner layers [36]. However, at RH > 80%, the water layer grows rapidly, with strong variations for very similar RH conditions [46]. Hence, subtraction of the accessible values for flat SiO_2_ (0.2 to 1 nm) from the lengths obtained from dynamic force spectroscopy, gives a lower limit of the water layer on TMV, which amounts to ≈1 nm (for RH < 80%). This value fits nicely with simplified models for water on nanoscale cylinders in the absence of condensation or evaporation phenomena (low vapor pressures) [31]. The disjoining pressure limits the water film thickness on a cylinder to (RA/γ)^1/3^, which is 1.1 nm for virion radius R = 9 nm, Hamaker constant A = 10^−20^ J and water surface tension γ = 0.07 N⋅m^−1^ (see also Section S7 of the Supporting Information).

## 3. Materials and Methods

Sample preparation: diluted TMV solutions in water (concentration: 0.05 mg/mL) were used in experiments. Droplets (40 μL) of TMV solutions were deposited on gold substrates or on freshly cleaved mica and left drying for 12 h. Flat gold surfaces (roughness: 0.4 nm) were prepared on commercial Silicon substrates (Silicon Valley Microelectronics, Santa Clara, CA, USA) by template stripping from annealed gold on mica (SPI Supplies, West Chester, PA, USA) [47]. Once prepared, they were cleaned by dipping in pure solvents (acetone, isopropanol) (Sigma-Aldrich (Merck Life Science), Madrid, Spain), then in milli-Q water, and, finally, dried. They were made hydrophilic before TMV deposition by incubation in oxygen plasma (8 min, 1 mbar) (Plasma System Type Femto, Diener Electronic, Ebhausen, Germany).

AFM imaging: AFM images were collected using a MFP3D microscope (Asylum Research, Santa Barbara, CA, USA) equipped with a cooling/heating stage for multifrequency AFM, and with an Agilent 5500 microscope (Keysight Technologies, Madrid, Spain) for single frequency experiments. Images at various RH levels were obtained by decreasing the temperature of the sample stage at a rate of 120°/min, and then waiting 30 min for thermal stabilization. Temperature in experiments was 25 °C (Figure 2a,b, Figure 3a,b), 20 °C (Figure 2c,d, Figure 3d,e), and 10 °C (Figure 2e,f, Figure 3g,h). Images were collected at a rate of 1 Hz and with 256 × 256 pixels. Rectangular cantilevers were used in experiments (Multi75Al BudgetSensors from Nanoandmore, Wetzlar, Germany), with nominal fundamental resonant frequency f_1_ = 75 kHz, spring constant k = 3 N/m, and tip radius R_0_ < 10 nm. Closed loop technology was applied for the scanning. Topography images were flattened, and profiles were analyzed with Gwyddion (www.gwyddion.net) and with WSxM [48]. Multifrequency (φ_2_) images (f_2_ ≈ 400 kHz) were collected simultaneously with topography. They were not processed, except for z-scale adjustments. Further details of the imaging conditions are reported in the Supporting Information.

Force and dissipated energy curves from AM-AFM force spectroscopy: for force reconstruction, only approach amplitude versus distance curves (APD curves) were used. The perfect overlap between the approach and the retracting path was verified with a drift < 0.5 nm. We used PPP-NCHR cantilevers with nominal fundamental resonance frequency f = 300 kHz, spring constant k = 15–30 N/m, and tip radius R_0_ < 10 nm (Nanosensors from Nanoandmore, Wetzlar, Germany). Sets of various APD curves (5–10 curves) were collected at the rate of 1.5 Hz in various humidity conditions and in dry samples. Dry samples (R < 10%) were obtained by flowing nitrogen in the environmental AFM chamber for 30 min at 25 °C (Figure 4a). Temperature of the sample stage during experiments was set to 25 °C (Figure 4b) and 10 °C (Figure 4c and Figure 5a) (P = 1 atm). Fast AM-AFM images were collected between sets of APD curves to verify the position of the TMV particles and that no sample damage had occurred. Further details of the dynamic force spectroscopy experiments are reported in the Supplementary Materials Section S2 and Section S6.

Determination of the sample RH%: we measured RH in the AFM chamber using a Fluke 971 hygrometer and adjusted the temperature T directly on the sample stage. The pressure P was constant (1 atm). From the definition of RH:

RH (T) = P/P_0_ (T) (2)

and from tabulated values of the vapor pressure P_0_ (T), we calculated RH for each adjusted temperature. Any value above 100% means saturation.

## 4. Conclusions

In conclusion we show that multifrequency AFM allows a clear visualization of water condensed between TMV virions, due to the improved contrast of water droplets at high humidity levels. The phase images of the second excited mode exhibit better contrast compared to the phase images relative to the first fundamental mode. Furthermore, dynamic force spectroscopy allows measuring the thickness of the water on TMV along the vertical direction, thus complementing the topographic information with an accurate height determination.

## Data Availability

All data are stored on internal servers at CIC nanoGUNE and are available upon request.

## Supplementary Materials

The following material is available online: Supporting Information pdf (with seven sections) including 11 figures, a table, and a simulation input file for the Surface Evolver freeware.

## Sample Availability

Samples of the compounds are not available from the authors.
