# Supplementary material for: Nanoscale Wetting of Single Viruses
Source: Molecules. 2021 Aug 26;26(17):5184. doi: 10.3390/molecules26175184 (PMC8434471; doi:10.3390/molecules26175184)
Supplement: Supplementary file 1 [file molecules-26-05184-s001.zip › molecules-1309918-supplementary.pdf]

Nanoscale Wetting of Single Viruses

Supporting information

Annalisa Calò [1,2,3,*], Aitziber Eleta-Lopez [3], Thierry Ondarçuhu [4], Albert Verdaguer [5] and Alexander M. Bittner [3,6,*]

1 Department of Electronic and Biomedical Engineering, University of Barcelona, Calle Marti i Fraquès 1-11, 08028 Barcelona, Spain; annalisa.calo@ub.edu
2 Institute for Bioengineering of Catalonia (IBEC), Calle Baldiri Reixac 10-12, 08028 Barcelona, Spain
3 CIC nanoGUNE (BRTA), Tolosa Hiribidea 76, 20018 Donostia, Spain; a.eleta@nanogune.eu
4 Institut de Mécanique des Fluides de Toulouse (IMFT)-Université de Toulouse, CNRS-INPT-UPS, 2 allée du professeur Camille Soula, 31400 Toulouse, France; thierry.ondarcuhu@imft.fr
5 Catalan Institute of Nanoscience and Nanotechnology (ICN2), CSIC and the Barcelona Institute of Science and Technology, Campus UAB, 08193 Bellaterra, Spain; averdaguer@icmab.es
6 Ikerbasque, Basque Foundation for Science, Pl. Euskadi 5, 48009 Bilbao, Spain, a.bittner@nanogune.eu
* Correspondence: annalisa.calo@ub.edu, a.bittner@nanogune.eu

Section 1. Experiments on mica surfaces

In addition to the data recorded on gold substrates, we carried out experiments also on mica surfaces, prepared simply by cleaving. Fig. S1 shows almost filled monolayers of side-by-side aligned virions on mica, with typical packing defects (TMV cannot form 3D crystals, but liquid crystals). The end-to-end assembly results in quasi infinitely long rods with the typical irregularly wrinkled surface. The surface coverage can be tuned with the concentration of the TMV suspension.

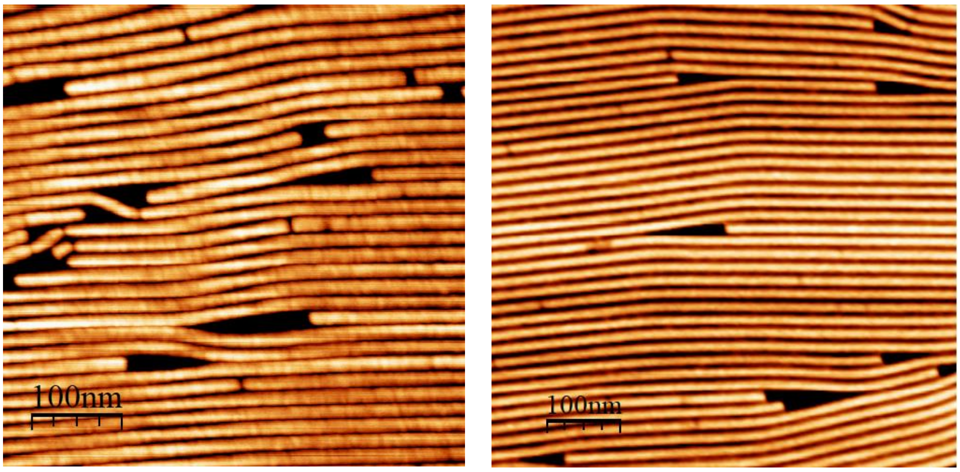

Fig. S1. AFM images of quasi-monolayers of TMV on mica at 10% RH (left) and at 70% RH (right).

Section 2. Details of multifrequency AFM experiments

Multifrequency imaging with the MFP3D microscope requires a careful setup of all parameters, related with the two excited frequencies. The first two flexural modes of the cantilever oscillation were excited by means of a linear combination of two sinusoidal voltages and separately detected[1]. The free amplitude of the second mode A_{02} was kept lower than that of the first mode[2], i. e. $A_{02} = 0.1 \cdot A_{01}$. We did not see any improvement in images by working at lower A_{02}. For the cantilevers used in this work (Multi75Al, Budget Sensors, $f_0 \sim 75$ kHz), the second resonant frequency was typically found at $f_2 \approx 400$ kHz. Free amplitudes A_{01} between 10 and 17 nm were used, setting the amplitude set-point A_1 at the highest value compatible with image quality. This procedure guarantees working in the net attractive tip-sample interaction regime for the fundamental mode. In these conditions, Φ_1 images showed z values always higher than 90°. Feedback gains were optimized to have the best contrast in Φ_2 images. Imaging rate was 1 Hz. Scan data from the second excited mode (A_2, Φ_2 maps) were collected simultaneously with sample topography and without feedback control.

In a typical experiment, first RH ≈ 10% was set, and topography and phase images were recorded for various A_L, A_{SP} values. The procedure was repeated for ≈ 60% and then for ≈ 90%, and finally repeated for ≈ 60%.

Φ_1 images are shown in fig. S2. They were collected simultaneously with images in Figure 2 of the main text. They often provide indirect information on the surface composition[3], and indeed in our case water droplets have a slightly different (5° to 10° lower) phase shift compared to the background. The improved contrast obtained in multifrequency AFM can be seen by comparing images in fig. S2a, S2b, S2c with those in Figure 2b, 2d, 2f of the main text.

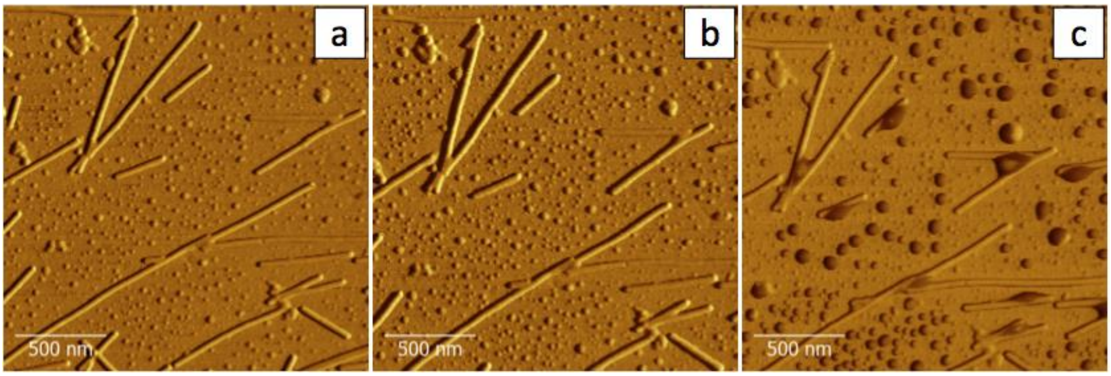

Fig. S2. Phase shift images of the first excited mode (Φ_1 images) corresponding to Figure 2 of the main text. Z-scale is 110°-135° (a), 90°-127° (b) and 90°-135° (c). RH is 56% (a), 76% (b) and > 100% (c). Resolution is 256 x 256 pixels.

In Φ_2 maps, the very large contrast ($\geq 10°$) allows distinguishing water for structures above ≈ 10 nm size. This high contrast is expected due to the sensitivity of the second mode, especially while operating the AFM in the non-contact regime, where long-range attractive forces dominate the tip-sample interaction[4,5]. In these conditions, differences of 1° to 2° have been predicted for Φ_2 in hydrocarbons with different chain lengths, and differences in the range of 10° are expected between water and mica, due to the different Hamaker constant[5]. Fig. S3 shows high resolution Φ_2 images corresponding to a detail of Figure 3 of the main text in a region where the two virions intersect. Here, very high contrast is observed around TMV contours at RH > 100%.

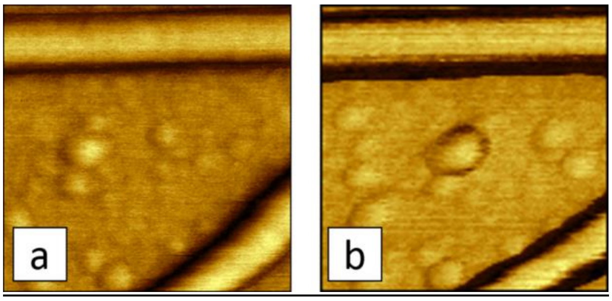

Fig. S3. High resolution Φ_2 images of two intersecting virions at RH = 56% (S3a) and > 100% (S3b) (see Figure 3 of the main text). Z-scale is 94°-105° (a) and 85°-96° (b). Image size is 230 x 230 nm and resolution 1.04 nm/pixel (a) and 1.9 nm/pixel (b).

The results shown in Figure 2 of the main text were reproduced on various parts of the samples. Obviously, the arrangement of the virions differs substantially. Fig. S4 shows two examples.

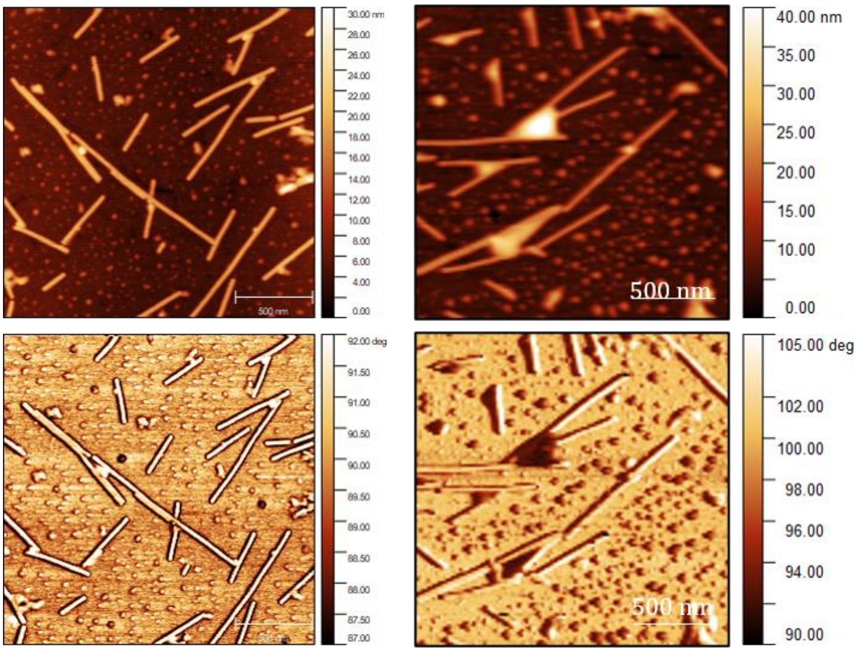

Fig. S4. AFM topographic images (top) and corresponding multifrequency $\Phi 2$ images (bottom) collected at RH = 56% (left) and > 100% (right).

Section 3. Substrate roughness

It must be noticed that a rough background has to be expected in the case of depositing biological samples on solid substrates, due to contaminations deriving from the purification process, or to the presence of stabilizing agents [6]. In our case, particles are present on the surface even at the lowest humidity levels (rms = 1.35 nm) (see Figure 1, 2, 3 of the main text). Due to their size, they could be short disks made up of coat proteins (34mers), or coat protein aggregates, with typical lateral size of 18 nm [7,8].

Section 4. Macroscopic and nanoscale contact angles

4.1. Macroscopic contact angles on Au surfaces

We determined the contact angles of water droplets on Au with a Krüss G10 goniometer on different samples. In Table S1 average contact angles on gold from 4 to 12 droplets are indicated at different surface pretreatment. Without any treatment, the high contact angle measured (~77°) indicates contamination from hydrophobic carbon or hydrocarbon species [9]. While Au is highly inert to oxidation by air, and has a very low catalytic activity at ambient conditions, laboratory air induces a contamination layer whose detection escapes many methods, including AFM. After oxygen plasma treatment, gold surfaces become hydrophilic and exhibit a maximum contact angle of 33° (the contact angle on freshly cleaved mica is <10°). After contact with TMV, the surfaces are covered to various degrees, depending on TMV concentration. From AFM data, 1 mg/ml TMV covers almost completely hydrophilic gold and mica surfaces. Hence the contact angle should refer in this case to a surface composed of pure TMV. In this case we measured a value of ~28°. This angle is practically identical to that on pure Au after 12 h contact to laboratory air, hence in the main text we use a value of 30° for the two surfaces under consideration. For low TMV coverage (0.001 mg/ml TMV), we expect a homogeneous, rough structure. The two surfaces of gold and TMV have the same contact angle, but the surface is now made up of 15 nm high TMV plus flat Au surface (rms: 0.1 nm). Despite lowering of the macroscopic contact angle is predicted in this case by the Wenzel theory [10], we measured a substantially higher contact angle of 60° in this case. This may be due to the pinning on isolated TMV particles acting as strong topographical defects, therefore increasing the contact angle. This effect is less pronounced at high coverage where collective effects decrease the pinning.

Au contact angles				
bare Au	Oxygen plasma cleaning	Laboratory exposure after plasma (12 h)	TMV 1 mg/mL (12 h)	TMV 0.001 mg/mL (12 h)
(77 ± 5)º	< 5º	(33 ± 4)º	(28 ± 2)º	(60 ± 5)º

Table S1. Macroscopic water contact angles measured on gold in various conditions.

4.2. Nanoscale contact angles

We determined local contact angles from AFM profiles at various positions. They correspond to the local slope of the liquid interface close to the three-phase lines, (virion-gold-water vapor or virion-liquid water-water vapor).

The cross-sections a1 and b1 (green) in fig. S5a and S5b refer to pure TMV on gold (or rather, to virions covered by an ultrathin layer of water). In this case, the angles represent an upper limit for contact angles because any further water condensation will result in structures that fill gap, spread on the surface and thus lower the contact angle. Indeed, here we found angles of 42° and 43° at both humidity values, larger than any other angle we measured.

Profiles a2, a4 and b2 (red) are used to determine the local contact angle of the droplet on the substrate surface. The measurements, more reliable on the larger droplets (fig. S5b and S5c). Profile b2 gives 36° for the nanoscale contact angle on gold, near to the macroscopic water/gold contact angle of 30°.

Finally, profiles a3 (only the positive angle of 25º), b3, b4, and b5 (light blue) allow us to estimate the water contact angle on the virion surface. Here, we have to consider that the virion surface, except for the very top, is not horizontal: The contact angle θ between a water droplet and a non-horizontal surface of a virion, is $\theta = \alpha + \beta$, where α is the measured tangent on the water droplet w.r.t the horizontal, and β is the angle that the virion surface makes with the horizontal (see fig. S5c). The value of 25° to 31° are well comparable to the 28° we found for water on a thin film of TMV (see above). Hence, also this contact angle shows no measurable deviation from the macroscopic value.

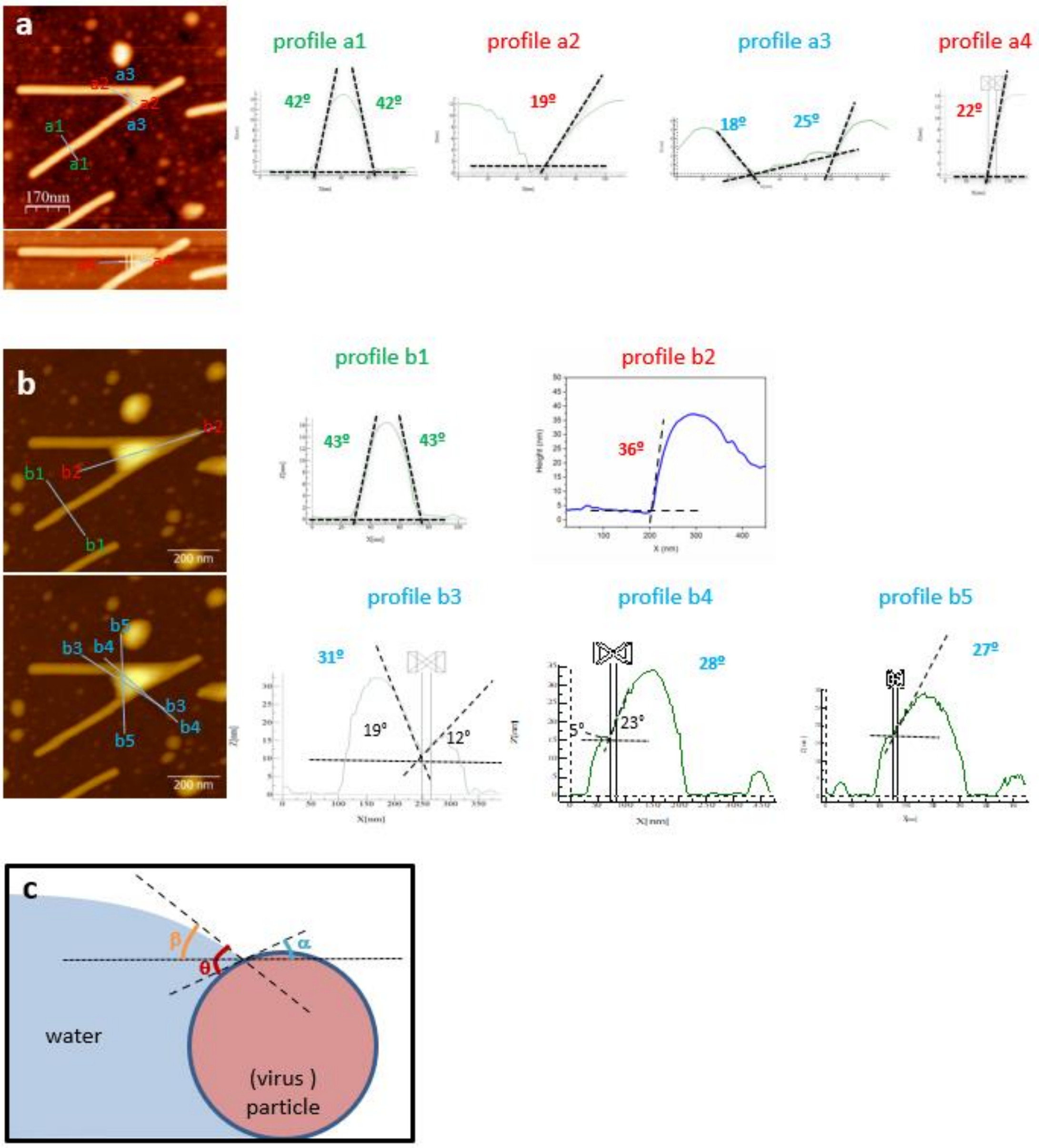

Fig. S5. AFM images and cross-sections (profiles) of a water droplet confined between two virions. (a) RH = 56%, (b) RH = 100%. Profiles a1 and b1 (green) are measured from the substrate to the virion sides, profiles a2, a4 and b2 (red) correspond to the contact angle of the water droplet on the substrate. Profiles a3, b3, b4, and b5 (light blue) are approaches to determine contact angles of water on the (non-horizontal) virion surface. The approach for profiles b3 and b4 is visualized in (c). Images (a) and (b) correspond to Figure 2a (a) and 2g (b) of the main text.

4.3. Corner wetting

The comparison is based on the nearly identical macroscopic contact angles of Au substrate and TMV, both 30° (see above). For simulations and theoretical considerations, this translates into a purely geometrically structured template. Experimentally, it is impossible to guarantee homogeneous structures, because any steep surface feature might feature chemical groups of density or even composition that differs from flat surfaces.

An interesting detail of adsorbed TMV is that its cross-section is slightly elliptical [11]. TMV on graphite has a circular cross-section, and is thus 18 nm high, with a very high contact angle between TMV and graphite close to 180°. TMV on gold is about 15 nm high, with a contact angle of ca. 120°, hence a wedge of 60° opening angle. It is exactly this wedge that we consider for corner wetting.

We determined virus widths from AFM cross-sections, as the FWHM of different profiles at various humidities. This gives (31 ± 2) nm at 56% RH, (30 ± 2) nm at 76% RH, and (35 ± 4) nm at RH > 100%. These values cannot correspond to the true diameter, rather tip convolution has to be taken into account. The apparent width of a dry virion should be $4 \cdot (R \cdot R_0)^{0.5}$, where R is TMV radius (9 nm) and R_0 the tip radius. This gives ~30 nm for a tip of R_0 about 6 nm. A slightly elliptical cross-section (for example, R = 10 nm instead of 9 nm) would have only a minor effect. Hence, starting from the three-phase line virion-gold-vapor, the side walls of the virions are filled, with water menisci of up to 2.5 nm thickness.

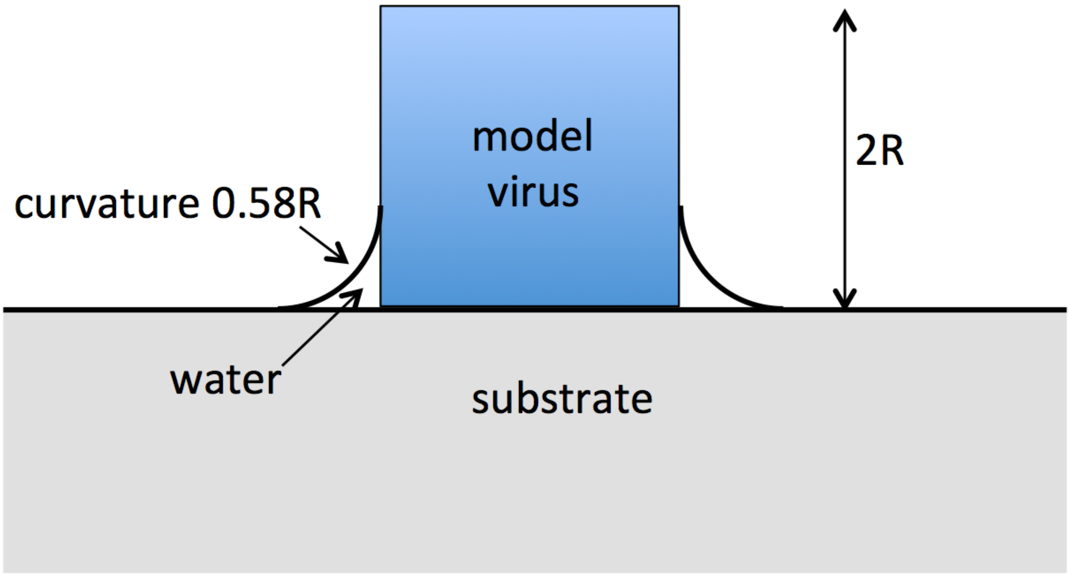

Fig. S6. Schematics of a water menisci at a 90° corner.

This phenomenon is well known from everyday experience, and detailed experiments [12] show that water wets a sharp edge much better than a flat surface, given a hydrophilic nature of the surface. For a 90° corner in a square-shaped cross-section of a capillary of width 2R, the radius (inverse curvature) of the liquid wedge is 0.58R (see fig. S6). Approximating the virus/gold edge as a 90° corner, this radius would be around 4 nm. The virus would thus appear not 18-20 nm wide, but 26 or 28 nm wide in this approximation. However, condensation or evaporation are neglected here, hence we would expect this to be valid for rather low humidities. This value is in good agreement with electron microscopy (wet STEM) data [11] obtained from adsorbed TMV at various humidities.

Multifrequency Φ2 images at high RH showed very strong contrast at the TMV contours (fig. S7). The width of the dark features from profiles enlarge from 2-4 nm at 56% RH to 5-8 nm at RH > 100% (see also fig. S3).

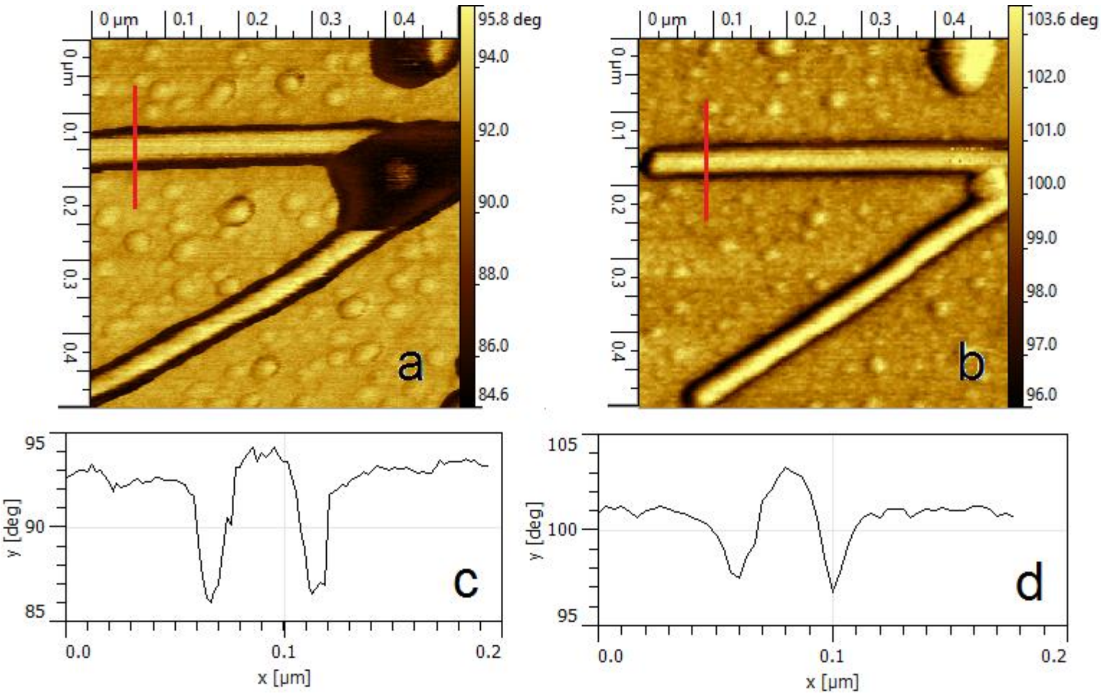

Fig. S7. Φ_2 details of TMV virions at RH 56% (a) and RH > 100% (b) and corresponding profiles (c, d).

Section 5. Details of the Surface Evolver simulations

The boundary conditions are a flat surface (gold) and two truncated cylinders, terminated at 2/3 (240°) of the complete circumference, to simulate the slightly flattened TMV particles. The contact angles on all surfaces were set to 30°, in analogy to the macroscopic measurements. The pressure was varied from -0.5 to 0.5 a. u., to simulate the filling of the gap both in low RH and oversaturated conditions.

The simulation file is provided below.

```
PARAMETER angle = 30    // interior angle between plane and surface,
degrees

PARAMETER angle_surf = 30 // surface contact angle

PARAMETER cyl_h = 0.5    // height of center of cylinder

gravity_constant 0  // start with gravity off

#define WALLT  (-cos(angle*pi/180))  // virtual tension of facet on
plane

#define SURFT  (-cos(angle_surf*pi/180))  // virtual tension of facet
on surface


constraint 1   /* the table top */
formula: x3 = 0

constraint 2   /* left */
formula: x2^2+(x3-cyl_h)^2 = 1

constraint 3   /* right */
formula: x1^2+x2^2+(x3-cyl_h)^2-1/(1+4)*(x1+2*x2)^2 = 1
//formula: x1^2+(x3-cyl_h)^2 = 1

constraint 4 /* end of right virus */
formula: x1+2*x2=0

constraint 5 /* end of right virus */
formula: x1+2*x2-30=0


constraint 6 /* bottom line of right virus*/
formula: .8660254*x1-x2+1.1337

boundary 3 parameters 1   //  left virus
x1:  0
x2:  cos(p1)
x3:  sin(p1)+0.5

boundary 4 parameters 1   //  left virus
x1:  13
x2:  cos(p1)
x3:  sin(p1)+0.5
```

```
vertices
1   1.6  1.5 0.0  constraint 1,2,3  /* 4 vertices on plane */
2   7.0  1.0 0.0  constraint 1,2
3   6.0  6.0 0.0  constraint 1,3
4   1.0  1.0 1  constraint 2,3
5   7.0  0.5 1  constraint 2
6   6.0  6.0 1  constraint 3

7   13.0  13.0 0  fixed   /* for table top */
8   13.0 -2.0  0  fixed
9   -2.0 -2.0  0  fixed
10  -2.0  13.0 0  fixed

11  -0.2 0.5 0.0        constraint 1,3,4   /* for right cylinder*/
12  -0.5 0.7 0.5        constraint 3,4
13  -0.2 0.5 1.366      constraint 3,4
14  0.2 -0.5 1.366      constraint 3,4
15  0.5 -0.7 0.5        constraint 3,4
16  0.2 -0.5 0.0        e     constraint 3,5
19  6.8 14.0 1.366      constraint 3,5
20  7.2 13.0 1.366      constraint 3,5
21  7.5 12.8 0.5        constraint 3,5
22  7.2 13.0 0.0        constraint 1,3,5

23  -pi/6   boundary 3  fixed  /* for left cylinder*/
24  0       boundary 3  fixed
25  pi/3    boundary 3  fixed
26 2*pi/3   boundary 3  fixed
27  pi      boundary 3  fixed
28 7*pi/6   boundary 3  fixed
29  -pi/6   boundary 4  fixed
30  0       boundary 4  fixed
31  pi/3    boundary 4  fixed
32 2*pi/3   boundary 4  fixed
33  pi      e     color yellow
3   3 7 -6 -9  tension WALLT constraint 3       color yellow
4   1  2  3 tension SURFT constraint 1          color yellow
5   4  5  6                                     color yellow

7  10 11 12  13  tension 0 fixed no_refine color red /* table top for
display */

8  14 25 -19 -24 tension 1 color brown /*  right cylinder*/
9  15 26 -20 -25 tension 1 color brown
10 16 27 -21 -26 tension 1 color brown
11 17 28 -22 -27 tension 1 color brown
12 18 29 -23 -28 tension 1 color brown

13 30 41 -35 -40  tension 1 color brown /*  left cylinder*/
14 31 42 -36 -41  tension 1 color brown
15 32 43 -37 -42  tension 1 color brown
16 33 44 -38 -43  tension 1 color brown
17 34 45 -39 -44  tension 1 color brown


bodies  /* one body, defined by its oriented faces */
1  1 2 3 4 5      pressure 0.5
2  8 9 10 11 12   e 13 14 15 16 17 volume 32.87
```

Section 6: Experimental details of the force reconstruction method

The cantilever resonant frequency f, Q factor and k were calibrated by means of the thermal noise method implemented in the microscope at tens of nm far from the surface. For force reconstruction experiments with the MFP3D, we found in general that an accurate calibration of the resonant frequency is required. Aberrant trends were observed in E_{diss} curves, even for small divergences of the working frequency from the exact f value in the proximity of the sample surface. More specifically, working at frequencies a few tens of Hz higher than the resonant value, E_{diss} starts to decrease in the region of mechanical contact (see fig. S8e, S8f), while at frequencies a few tens of Hz lower than the resonant value, an inflection towards negative E_{diss} values was observed in the long-range (fig. S8c). This behavior is independent on the substrate. We calibrated the working frequency by means of the same reconstructed F and E_{diss} curves on top of model SiO_2 and BaF_2 substrates (see fig. S8).

The working frequency was chosen as the value for which E_{diss} starts to increase when F starts to show a distance-dependent decay in the long range (fig. S8a and S8d). Even though numerical simulations often show E_{diss} rapidly increasing [13] approximately starting from F_{ad}, a dissipation of energy has to be expected already in the attractive regime [14,15]. For the cantilevers used in this work (PPP-NCHR, Nanosensors, nominal f = 300 kHz), these values were systematically 30-50 Hz lower than those determined at a distance of 5 μm from the sample surface, by completely retracting the piezo actuator after approach.

The conversion of A from volts to nm was performed by means of the same code implemented for force reconstruction, using the method reported by Gadelrab and coauthors [16]. Tip radius was also monitored *in situ*, by checking that the required A_0 to achieve a smooth transition between the attractive and the repulsive regime did not vary during experiments [17]. In the case of TMV and for new cantilevers (nominal R_0 < 10 nm), A_0 = 27 nm guarantees observing a smooth transition in APD curves collected within a vertical range of 20 nm, when the amplitude is reduced of 10% of its free value. For blunt tips, the variation in the tip radius (about 40%) was estimated from the value of the (higher) free amplitude necessary to observe smooth transitions in APD curves (A_0 =36 nm) [17], as:

$$R = R_0 \cdot \left(\frac{A_1}{A_0}\right)^{1.1} \qquad \text{Eq. S1}$$

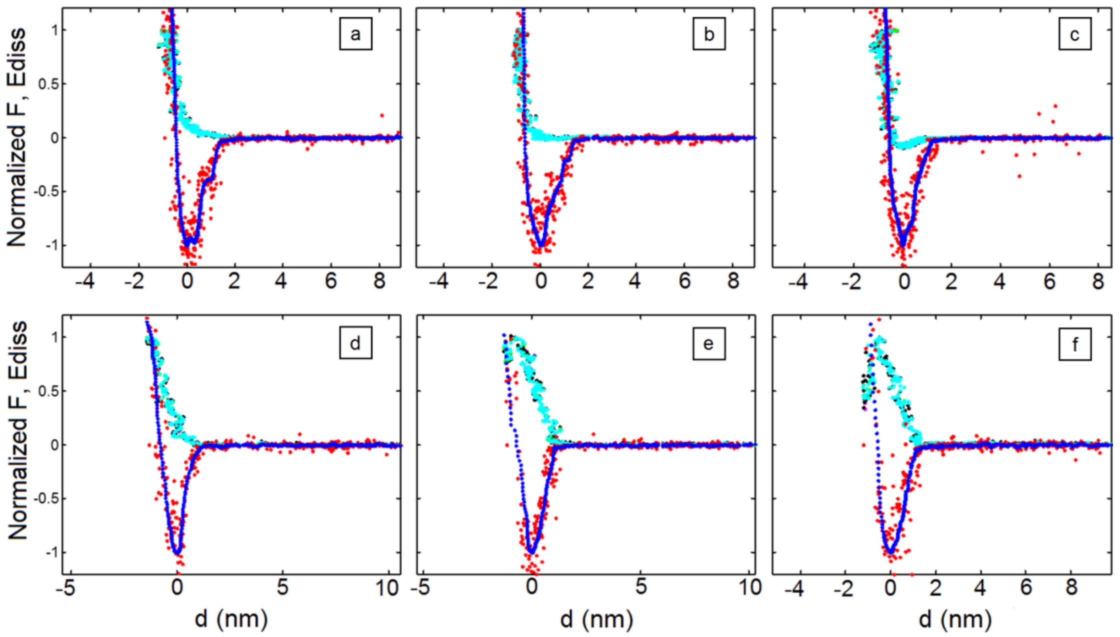

Fig. S8. Normalized reconstructed F (red dots: raw data; blue line: smoothed data) and E_{diss} (black dots: raw data; cyan dots: smoothed data) curves collected onto a freshly cleaved BaF_2 (a, b, c) and a clean SiO_2 surface (d, e, f) at f (a, d), f -20 Hz (b), f - 30 Hz (c), f + 20 Hz (e), f + 30 Hz (f). A_0 = 36 nm (a, b, c) and 22.5 nm (d, e, f). F_{ad} = 6.6 nN (a), 5.9 nN (b), 7.9 nN (c), 2.3 nN (d), 2.5 nN (e) and 1.8 nN (f). E_{max} = 241 eV (a), 262 eV (b), 294 eV (c), 45 eV (d), 55 eV (e) and 44 eV (f). Curved were collected in dry conditions (RH < 10%).

APD curves showing smooth transitions were converted into F and E_{diss} vs. minimum tip-sample separation distance *d* by means of a code implemented in Matlab [13]. Here, the Sader-Jarvis-Katan's equation was numerically integrated and the energy dissipated per oscillation cycle (E_{diss}) calculated according to Cleveland's equation [18]:

$$E_{diss} = \frac{\pi \cdot k \cdot A_0 \cdot A}{Q}\left[sin(\varphi) - \frac{A}{A_0}\right] \qquad \text{Eq. S2}$$

Smoothing of the curves was obtained by means of the same code implemented in Matlab [19,20].

6.1. Force reconstruction for a model flat SiO_2 surface

Preliminary tests were performed on hard substrates before collecting APD curves on top of TMV particles. The conservative tip-sample interaction force was reconstructed from APD curves collected at A_0 = 22.5 nm.

Fig. S9a and S9b show an example of approach APD curves on a dry SiO_2 surface (RH < 10%). The corresponding reconstructed force curve is shown in fig. S5c (blue curve). Curves like the ones shown in fig. S5a and S5b exhibit a smooth transition between the net attractive and the repulsive tip-sample interaction regime [21]. This is an important requirement for performing humidity-sensitive measurements. In these conditions, in fact, the whole tip-sample interaction force can be recovered in force reconstruction routines without discontinuities, from long-range attractive forces to short-range repulsion, or mechanical contact [22]. This feature of dynamic AFM measurements constitutes an improvement with respect to static force spectroscopy. Here, the ubiquitous jump to contact instability prevents the access to the region of long-range forces and the study of their distance dependence in many cases [19]. Dynamic force curves obtained at the smooth transition definitely permit to study wetting at the nanoscale, which gives rise to long range, attractive forces that act a few nm before the region of mechanical contact and of consequent sample deformation.

The force profile shown in fig. S9c indicates that in dry conditions the net attractive regime can be quantified by the Hamaker constant, as it is customary in dynamic AFM [13]. The decaying region in the force curve, in fact, spans a vertical distance ≤ 1 nm and shows a distance evolution that can be approximated with a power law decay, i. e. $F \propto d^{-2}$. After the absolute minimum (d = 0), the force starts to increase rapidly with distance and its profile becomes vertical. In this region short range repulsion and mechanical deformation are the dominant tip-sample interaction [13]. The evolution of the dissipated energy per cycle is also reported in fig. S9c (red curve). A monotonically increasing evolution of E_{diss} vs. d is observed on SiO_2 surfaces. E_{diss} starts to increase at a distance approximately corresponding to the onset of the power-law decay in F.

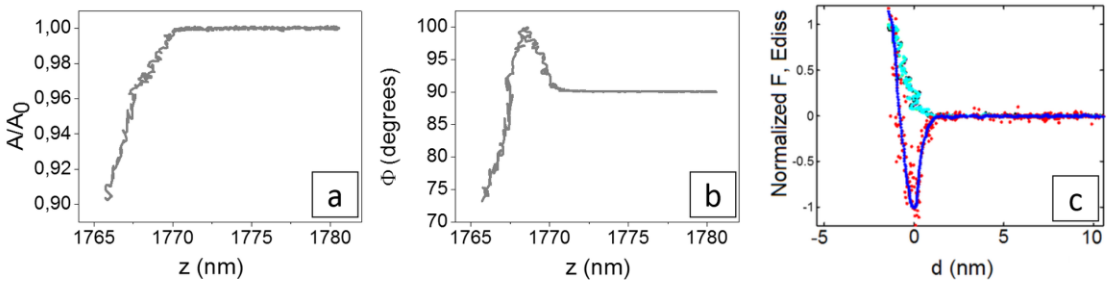

Fig. S9. Amplitude (a) and phase shift (b) vs. z-piezo position curves collected on top of a dry SiO_2 substrate (RH < 10%). (c) Corresponding normalized reconstructed force (red dots: raw data; blue line: smoothed data) and dissipated energy (black dots: raw data; cyan dots: smoothed data) curves. F_{ad} = 2.3 nN, E_{max} = 45 eV.

6.2. F and E_{diss} profiles in the case of blunt tips

Interesting features come out when tips are not ultrasharp. We observed that the thickness of water layers on TMV (h_{TMV}) are crucially dependent on the probe size. Increasing the tip radius, curves showed higher adhesion forces (F_{ad}) and

maximum dissipated energies (E_{max}), a fact that is expected due to the dependence of dynamic observables, and mainly A_0, on the probe geometry [13,17].

In high RH conditions, force curves on TMV showed square well-like profiles starting at $d \sim 6$ nm from the contact, when the probe size increases of about 40%. This would mean R ~ 14 nm, if the minimum R_0 corresponds to the nominal value of 10 nm. E_{diss} profiles also showed a well-defined, step-like increase in correspondence of this distance (see fig. S10b and Figure 5a of the main text). Tip size can affect the magnitude of the attractive force in the long-range regime [13]. At the same time, wetting phenomena could be amplified when dealing with blunt probes. They may include scenarios where the dynamic formation of a capillary neck is better described by instabilities, like capillary condensation and van der Waals attractive forces, that produce the formation of capillary necks at distances higher than 2h (h being the thickness of water layers on the tip and on the imaged surface) [23,24], i. e. at d_{on} = 3h.

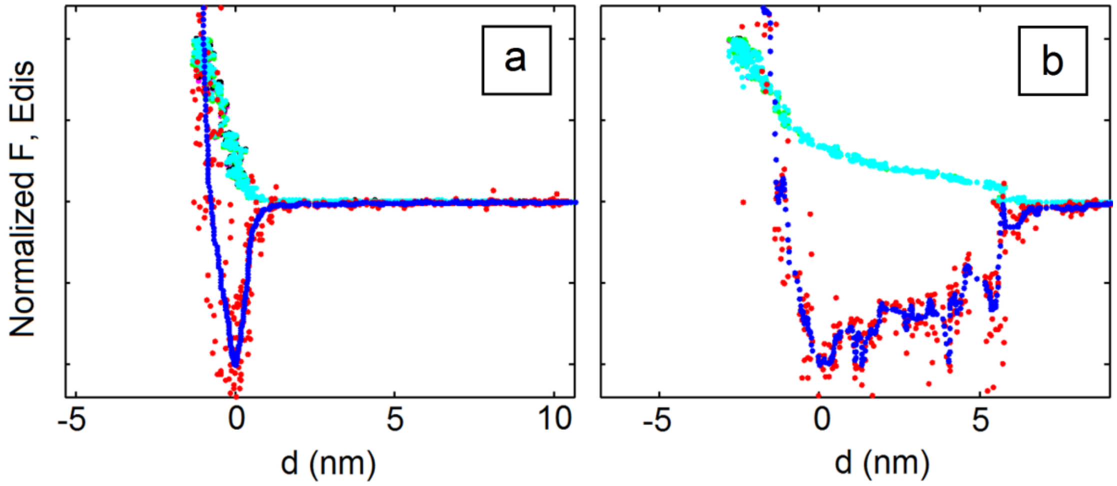

Fig. S10. F (red dots: raw data; blue line: smoothed data) and E_{diss} (tip in dry (RH < 10%) (a) and in wet conditions (RH > 100%) (b). F_{ad} = 3.4 nN, E_{max} = 167 eV (a), F_{ad} = 2.6 nN, E_{max} = 276 eV (b). Here, the abrupt jump in E_{diss} at $d = d_{on}$ is clear. A_0 = 36 nm.

In the case of small tips, the energy evolution around d_{on} suggests a small amount of energy is involved in the formation of the capillary neck (see Figure 4c in the main text). In this condition, abrupt attractive forces or capillary condensations that would affect energy dissipation can be considered negligible. Also, water menisci could form upon simple geometrical contact of the two hydrated surfaces of the tip and the TMV sample, a fact that also would impact on the amount of energy dissipated in the formation of a nanoscale tip-water-TMV contact [19,23].

Fig. S10a shows the corresponding curves obtained with blunt tips in dry conditions. Here, the distance covered from the onset of the attractive region till contact is similar to that obtained with ultrasharp tips, 1.2 ± 0.4 nm. Also, the force evolution is similar to the one shown in fig. S9c. It has to be noticed that this distance approximates the limits of the vertical resolution for dynamic force spectroscopy measurements. For this reason, in some cases it could be hard to

distinguish cases were very thin water layers (0.5-1 nm thick) are confined between the tip and the surface, from cases of completely dry surfaces where van der Waals attraction between surface atoms only contributes to the long range evolution in reconstructed force curves.

Section 7: Comparison of the observed thin water film with simple wetting scenarios

7.1. Water films on a cylinder

Droplets on hydrophilic flat surfaces are supposed to produce extended areas of extremely thin wetting films [12,25-29]. These films are exceptionally difficult to detect for water, so usually other liquids are employed. On a solid cylinder of radius R and Hamaker constant A, the disjoining pressure limits the film thickness[28] of a liquid of surface tension γ to $(RA/\gamma)^{1/3}$. For water on nanoscale cylinders or tubes with typical Hamaker constants, this thickness is ≈ 1 nm for $A=10^{-20}$ J, as we already discussed in the main text. An additional feature of this simple theory is worth mentioning: the film should become unstable above $(3R^2A/\gamma)^{1/4} \approx 2.4$ nm thickness. We were not able to trace this critical thickness by dynamic spectroscopy, but our data clearly show that oversaturation can create much thicker films, which indeed are unstable, and break up into droplets. In fig. S11 an example is shown for a single virion.

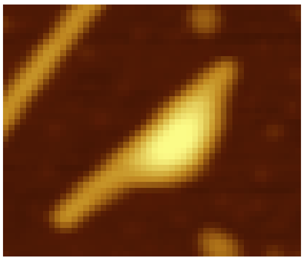

Fig. S11. AFM detail showing condensed water on a virion. Z-scale is 30 nm, image size is 300x300 nm^2 (40x40 pixels).

7.2. Water films on rectangular posts

Rectangular posts of microdimensions[29], investigated theoretically and with experiments, can be compared to virions of equal width and height z. However, the vertical walls of the posts are not wettable, only the top surface, which provides a confinement that is not present for viruses. But even if this were present, only a liquid film should form on top of the virions, and no bulge or droplet, as we also find. The minimum volume for the existence of a bulge is $V \approx x \cdot z^2/3 = 75 \cdot x\ (nm)^2$, which is very far above any volume we observed on the virions. An exception are the bulges/droplets found at the virions (see fig. S10). As

calculated, the volumes are here very high, in the range of $200 \cdot x$ $(nm)^2$, x being the axial coordinate.

Bibliography

1. Proksch, R. Multifrequency Repulsive-Mode Amplitude Modulated Atomic Force Microscopy. *Appl. Phys. Lett.* **2006**, *89*, 113121.

2. Damircheli, M.; Payam, A. F.; García, R. Optimization of Phase Contrast in Bimodal Amplitude Modulation AFM. *Beilstein J. Nanotechnol.* **2015**, *6*, 1072-1081.

3. García, R.; Magerle, R.; Perez, R. Nanoscale Compositional Mapping with Gentle Forces. *Nat. Mater.* **2007**, *6*, 405-411.

4. Lozano, J. R.; García, R. Theory of Multifrequency Atomic Force Microscopy. *Phys. Rev. Lett.* **2008**, *100*, 076102.

5. Rodríguez, T. R.; García, R. Compositional mapping of surfaces in atomic force microscopy by excitation of the second normal mode of the microcantilever. *Appl. Phys. Lett.* **2004**, *84*, 449-451.

6. Calò, A.; Sanmartí Espinal, M.; Iavicoli, P.; Persuy, M.-A.; Pajot Augy, E.; Gomila, G.; Samitier, J. Diffusion-Controlled Deposition of Natural Nanovesicles Containing G-Protein Coupled Receptors for Biosensing Platforms. *Soft Matter* **2012**, *8*, 11632-11643.

7. Mueller, A.; Eber, F. J.; Azucena, C.; Petershans, A.; Bittner, A. M.; Gliemann, H.; Jeske, H.; Wege, C. Inducible Site-Selective Bottom-Up Assembly of Virus-Derived Nanotube Arrays on RNA-Equipped Wafers. *ACS nano* **2011**, *5*, 4512-4520.

8. Eber, F. J.; Eiben, S.; Jeske, H.; Wege, C. RNA-Controlled Assembly of Tobacco Mosaic Virus-Derived Complex Structures: from Nanoboomerangs to Tetrapods. *Nanoscale* **2015**, *7*, 344-355.

9. Mittal K. L. *Advances in Contact Angle, Wettability and Adhesion*, Vol. 1, Wiley, **2013**.

10. Herminghaus, S.; Brinkmann, M.; Seemann, R. Wetting and Dewetting of Complex Surface Geometries. *Annu. Rev. Mater. Res.* **2008**, *38*, 101-121.

11. Alonso, J. M.; Tatti, F.; Chuvilin, A.; Mam, K.; Ondarçuhu, T.; Bittner, A. M. The Condensation of Water on Adsorbed Viruses. *Langmuir* **2013**, *29*, 14580-14587.

12. Dong, M.; Chatzis, I. The Imbibition and Flow of a Wetting Liquid Along the Corners of a Square Capillary Tube *J. Coll. Interf. Sci.* **1995**, *172*, 278-288.

13. Santos, S.; Amadei, C. A.; Verdaguer, A.; Chiesa, M. Size Dependent Transitions in Nanoscale Dissipation. *J. Phys. Chem. C* **2013**, *117*, 10615-10622.

14. Martinez, N. F.; García, R. Measuring Phase Shifts and Energy Dissipation with Amplitude Modulation Atomic Force Microscopy. *Nanotechnology* **2006**, *17*, S167-S172.

15. Gadelrab, K. R.; Santos, S.; Souier, T.; Chiesa, M. Disentangling Viscosity and Hysteretic Dissipative Components in Dynamic Nanoscale Interactions. *J. Phys. D: Appl. Phys.* **2012**, *45*, 012002.

16. Gadelrab, K. R.; Santos, S., Chiesa, M. Heterogeneous Dissipation and Size Dependencies of Dissipative Processes in Nanoscale Interactions. *Langmuir*, **2013**, *29*, 2200-2206.

17. Santos, S.; Guang, L.; Souier, T.; Gadelrab, K.; Chiesa, M.; Thomson, N. H. A Method to Provide Rapid in Situ Determination of Tip Radius in Dynamic Atomic Force Microscopy. *Rev. Sci. Instrum.* **2012**, *83*, 043707.

18. Cleveland, J. P.; Anczykowski, B.; Schmid, A. E.; Elings, V. B. Energy Dissipation in Tapping-Mode Atomic Force Microscopy. *Appl. Phys. Lett.* **1998**, *72*, 2613-2615.

19. Calò, A.; Vidal Robles, O.; Santos, S.; Verdaguer, A. Capillary and Van der Waals Interactions on CaF_2 Crystals from Amplitude Modulation AFM Force Reconstruction Profiles under Ambient Conditions. *Beilstein J. Nanotechnol.* **2015**, *6*, 809-819.

20. Calò, A.; Domingo, N.; Santos, S.; Verdaguer, A. Revealing Water Films Structure from Force Reconstruction in Dynamic AFM. *J. Phys. Chem. C* **2015**, *119*, 8258-8265.

21. Garcia, R.; San Paulo, A. Amplitude Curves and Operating Regimes in Dynamic Force Microscopy. *Ultramicroscopy* **2000**, *82*, 79.

22. Amadei, C. A.; Santos, S.; Pehkonen, S. O.; Verdaguer, A.; Chiesa, M. Minimal Invasiveness and Spectroscopy-Like Fingerprints for the Characterization of Heterogeneous Nanoscale Wetting in Ambient Conditions. *J. Phys. Chem. C* **2013**, *117*, 20819-20825.

23. Barcons, V.; Verdaguer, A.; Font, J.; Chiesa, M.; Santos, S. Nanoscale Capillary Interactions in Dynamic Atomic Force Microscopy. *J. Phys. Chem. C* **2012**, *116*, 7757-7766.

24. Yaminsky, V. V. The Hydrophobic Force: the Constant Volume Capillary Approximation. *Colloids Surf. A Physicochem. Eng. Asp.* **1999**, *159*, 181-195.

25. Zhong Y.; Jacobi, A. M.; Georgiadis, J. G. Effects of Surface Chemistry and Groove Geometry on Wetting Characteristics and Droplet Motion of Water Condensate on Surfaces with Rectangular Microgrooves. *Int. J. Heat Mass Transf.* **2013**, *57*, 629-641.

26. Seeman, R.; Brinkmann, M.; Kramer, E. J.; Lange, F. F.; Lipowsky, R. *Proc. Natl. Acad. Sci. U.S.A.* **2005**, *102*, 1848-1852.

27. Vahid, A.; Moosavi, A. Morphology of Nanodroplets on Structured Surfaces. *J. Phys. D: Appl. Phys.* **2013**, *46*, 215302.

28. Mattia, D.; Starov, V.; Semenov, S. Thickness, Stability and Contact Angle of Liquid Films on and inside Nanofibres, Nanotubes and Nanochannels, *J. Coll. Interf. Sci.* **2012**, *384* 149-156.

29. Ferraro, D.; Semprebon, C.; Tóth, T.; Locatelli, E.; Pierno, M.; Mistura, G.; Brinkmann, M. Morphological Transitions of Droplets Wetting Rectangular Domains. *Langmuir*, **2012**, *28*, 13919-13923.
